# *Streptococcus halichoeri*: Comparative Genomics of an Emerging Pathogen

**DOI:** 10.1155/2020/8708305

**Published:** 2020-02-18

**Authors:** Kirsi Aaltonen, Ravi Kant, Marjut Eklund, Mirja Raunio-Saarnisto, Lars Paulin, Olli Vapalahti, Thomas Grönthal, Merja Rantala, Tarja Sironen

**Affiliations:** ^1^Department of Veterinary Biosciences, Faculty of Veterinary Medicine, University of Helsinki, Helsinki, Finland; ^2^Department of Virology, Faculty of Medicine, University of Helsinki, Helsinki, Finland; ^3^Department of Equine and Small Animal Medicine, Faculty of Veterinary Medicine, University of Helsinki, Helsinki, Finland; ^4^Finnish Food Authority, Seinäjoki, Finland; ^5^Institute of Biotechnology, University of Helsinki, Helsinki, Finland; ^6^HUSLAB, Hospital District of Helsinki and Uusimaa, Helsinki, Finland

## Abstract

Streptococcus halichoeri is an emerging pathogen with a variety of host species and zoonotic potential. It has been isolated from grey seals and other marine mammals as well as from human infections. Beginning in 2010, two concurrent epidemics were identified in Finland, in fur animals and domestic dogs, respectively. The fur animals suffered from a new disease fur animal epidemic necrotic pyoderma (FENP) and the dogs presented with ear infections with poor treatment response. S. halichoeri was isolated in both studies, albeit among other pathogens, indicating a possible role in the disease etiologies. The aim was to find a possible common origin of the fur animal and dog isolates and study the virulence factors to assess pathogenic potential. Isolates from seal, human, dogs, and fur animals were obtained for comparison. The whole genomes were sequenced from 20 different strains using the Illumina MiSeq platform and annotated using an automatic annotation pipeline RAST. The core and pangenomes were formed by comparing the genomes against each other in an all-against-all comparison. A phylogenetic tree was constructed using the genes of the core genome. Virulence factors were assessed using the Virulence Factor Database (VFDB) concentrating on the previously confirmed streptococcal factors. A core genome was formed which encompassed approximately half of the genes in Streptococcus halichoeri. The resulting core was nearly saturated and would not change significantly by adding more genomes. The remaining genes formed the pangenome which was highly variable and would still evolve after additional genomes. The results highlight the great adaptability of this bacterium possibly explaining the ease at which it switches hosts and environments. Virulence factors were also analyzed and were found primarily in the core genome. They represented many classes and functions, but the largest single category was adhesins which again supports the marine origin of this species.

## 1. Introduction

*Streptococcus halichoeri* was first described in 2004. It was isolated from grey seals (Halichoerus grypus) wherein it derives its name. The bacteria were found from wounds that had been inflicted by other seals, but evidence of systemic infection was also found [1]. *S. halichoeri* is one of only three Streptococcus species associated with marine mammals; the other two are *Streptococcus phocae* and *Streptococcus iniae*; the latter has also been found in farmed marine aquacultures and humans and has a significant pathogen potential [1]. *S. halichoeri* was found to be Gram-positive and belonging to the Lancefield group B. They are cocciforms that grow in pairs or short chains. In addition, they are nonhemolytic, facultatively anaerobic, and catalase-negative [1].

*S. halichoeri* has subsequently been found in humans in 2014 in a man with postoperative empyema [2] and in a diabetic man with infectious cellulitis [3]. In addition, several isolates have been obtained from human blood of both septicemic patients and others with unknown symptoms in the United States of America. The bacterium is hence considered an emerging pathogen that can cause serious disease in humans [4]. Subsequently, this bacterium has also been found in Steller sea lion (*Eumetopias jubatus*) [5] and the European badger (*Meles meles*) in which it was found to cause serious clinical symptoms [6].

During 2007, a novel and severe disease emerged in fur animals and was named “fur animal epidemic necrotic pyoderma” (FENP). The pathogen most strongly associated with the disease was *Arcanobacterium phocae* [7] previously only detected in marine mammals. The source of the original host shift of *A. phocae* is thought to have been infected seal meat used as feed [8]. During the investigation of the FENP outbreak, a Streptococcus species, previously undetected in fur animals, was also found in many samples, especially from mink. It was unclear whether this *Streptococcus* spp. together with a few other bacteria contributed to the disease [7]. The Streptococcus was later identified as *Streptococcus halichoeri*. While investigating the FENP epidemic, it has been found additionally in a quality control sample of herring from the Gulf of Finland used to prepare feed for the mink (unpublished observation). At the same time period as the outbreak of FENP, Finnish pet dogs were afflicted with an ear infection with poor treatment response and *S. halichoeri* was isolated from samples of the diseased dogs. Later on, this bacterium was also isolated from skin infections of dogs. These isolates were characterized by Eklund and colleagues [9] using conventional bacteriological, biochemical, and sequencing methods.

Our study focuses on further characterizing this emerging pathogen through sequencing the whole genomes of 20 isolates. This approach permits analysis of the core genome and virulence factors of *S. halichoeri*, allows more reliable phylogenetic analyses and attempts to trace the direction and frequency of previous bacterial introductions and (cross-species) transmissions.

## 2. Materials and Methods

### 2.1. Bacterial Isolates, Growth Conditions, and DNA Extraction

The bacterial strains (*n* = 20) included in this project have been characterized by Eklund et al., and the selection of strains was based on clustering in PFGE [9]. Ten isolates were from canine infections, five from mink, two from Finnraccoon, and one from a blue fox. The canine isolates are all from diagnostic samples of superficial or deep pus from cases of otitis or dermatitis. The clinical significance is unclear as all of these findings were of mixed culture most often together with *Staphylococcus pseudintermedius.* The eight fur animal isolates are all from severe dermatitis lesions. Findings from Nordgren et al. [10] suggest a possible role for *S. halichoeri* in the pathogenesis of FENP. Also, two reference strains were included, one from a seal (CCUG 48324) [1] and one from human isolate (CCUG 67100) [4]. The origin of the strains and their characteristics are listed in Table 1.

The bacteria were grown on blood agar plates with 4% defibrinated sheep blood, overnight. A single colony was then inoculated into 2 ml of Super Broth medium. They were grown at +37°C with mild shaking for 24 hours and harvested by centrifugation for 5 minutes at 4,500 g. The cells were stored in -20°C awaiting extraction. The DNA was extracted using the Epicentre by Lucigen MasterPure Gram Positive DNA Purification Kit (Lucigen Corp., Wisconsin, USA) according to the kit instructions. An overnight lysozyme treatment, stated optional in the kit, was used to ensure bacterial lysis.

### 2.2. Genome Sequencing and Annotation

Genomes of the 20 *S. halichoeri* isolates were sequenced at the Institute of Biotechnology (University of Helsinki, Finland) using next-generation sequencing platforms. Genomic DNA (0.5 mg) was sheared using a Bioruptor NGS Sonicator (Diagenode) to approximately 600 bp fragments. The fragments were polished, A-tailed, and ligated to a TruSeq truncated adapter. Purification of the ligation reaction was done using AMPure XP beads (Agencourt, Beckman Coulter). PCR of the libraries were done using Phusion Hot Start II DNA Polymerase (Thermo Fisher) and index P7 primers and full-length P5 adapter primers. The reactions were pooled and purified with AMPure XP beads. Size selection of the pool was done according to Lundin et al. [11]. The obtained library pool was paired-end sequenced on a MISeq Sequencer using the v3 600 cycle kit (Illumina).

Genomes of the 20 newly sequenced *S. halichoeri* strains were deposited in GenBank under the accession numbers listed in Table 1. The annotation was performed using the assembled DNA sequences of the 20 new draft genomes from these isolates. The genomes were run through an automatic annotation pipeline RAST (Rapid Annotation using Subsystem Technology) [12], followed by manual curation in few cases.

### 2.3. Orthologous Gene Prediction and Genome Sequence Comparison

Identification of orthologous genes for 20 *S. halichoeri* genomes was performed by an all-against-all comparison of the genes of all genomes using blastp [13] with the standard scoring matrix BLOSUM62 and an initial *E*-value cut-off of 1*e*^−05^. The bit score of every blast hit was set into proportion to the best bit score possible, the bit score of a hit of the query gene against itself. The outcome for this was a score ratio value (SRV) between 0 and 100 that reflected the quality of the hit much better than the raw blast bit score [14].

Two genes were acknowledged orthologous if a reciprocal best blast hit existed among them, and both hits had an SRV > 32. The SRV threshold is computed from distribution of blast hits between analyzed sequences as described in the supplement of Blom et al. [15]. Based on this orthology principle, the core genome was calculated as the set of genes that had orthologous genes in all other analyzed strains.

The pangenome was estimated as the set of all unique genes of a set of genomes. All genes of one reference genome were considered the basic set for the calculation. Afterwards, the genes of a second genome were matched with this set, and all genes in the second genome that had no orthologous gene in the starting gene set were added to this set. This process was iteratively repeated for all genomes of the compared set, leading to the pangenome. The circular plot comparing 20 genomes was generated with BioCircos [16].

### 2.4. Phylogenetic Construction

The phylogenetic tree was calculated using a somewhat modified version of the pipeline proposed by Zbodnov and Bork [17]. Alignments of each core gene set are compiled using MUSCLE [18], the numerous resulting multiple alignments were concatenated, and poorly aligned positions were removed using GBLOCKS [19]. The trimmed multiple alignment was used to create a phylogenetic tree using the neighbour-joining operation of PHYLIP [20].

### 2.5. Identification of the Putative Virulence Factors in the Genomes

The Virulence Factor Database (VFDB) [21] as well as known virulence factors of Streptococci was used as guidelines when choosing the putative virulence factors to be sought. The Virulence Factor Database is based on experimentally validated or strongly suspected bacterial virulence factors from multiple bacterial species. There are listed known virulence factors from twenty different species of streptococci encompassing 56 different strains with 75 recognized virulence factors. The closest genetic relative to *S. halichoeri* in this database is *Streptococcus agalactiae* which also correlated with the identified virulence factors. Two different approaches were used to analyze these factors, utilizing either the core genome or the annotated pangenome. This enabled recognition of the virulence factors with most importance as well as the more dispensable ones.

## 3. Results and Discussion

### 3.1. General Features of the Genomes of 20 *Streptococcus halichoeri* Isolates

In this study, we have constructed sequences of 20 *S. halichoeri* isolates via high-throughput sequencing. The assembled draft sequences were initially annotated using an automated pipeline for gene identification and then afterwards improved by additional manual curation. Plasmid DNA sequences were excluded from this annotation process. A list of the annotated genes predicted for the 20 newly sequenced genomes is given as supporting information ([Supplementary-material supplementary-material-1]), with each genomic sequence deposited into GenBank (Table 1). The general features of the 20 new *S. halichoeri* genomes included and analyzed in this study are presented in Table 1. Here, genomes were characterized from dog [10], mink [5], Finnraccoon [2], human [1], blue fox [1], and seal [1] hosts. Till date, there are no other *S. halichoeri* genome sequences present in the NCBI RefSeq database making this study the first genomic study of *S. halichoeri*.

Despite the fact that all 20 genomes are draft assemblies, they still represent good quality sequence data for performing genomic comparisons ([Supplementary-material supplementary-material-1]). The average coverage of the genome sequencing ranges widely from 61-fold (S212) to 280-fold (CCUG48324). Furthermore, the number of contigs in the assembled genomes was between 32 and 244 (P1033 and P399, respectively). The genome size was ranging between 1.89 (CCUG48324) and 2.26 (P399) Mbps. The total GC content varies only slightly and ranged between 41.2 and 41.8%. The numbers of predicted protein-encoding open-reading frames (ORFs) in the 20 isolates varied from 1,873 (CCUG48324) to 2,198 (P399) suggesting reasonable diversity in the species of *S. halichoeri*.

### 3.2. Phylogeny

Pangenomic studies are usually performed without referencing the individual ecological niches the isolates are derived from. However, the host source of the bacterial strains should be considered an essential parameter for the pangenome to be deciphered correctly. Reconstructing a core genome-based phylogenic tree from our 20 *S. halichoeri* strains offers additional understanding between the incidental phyletic associations and of any common origins by presenting possible correlations. Comparison of the 20 genomes illustrated in [Supplementary-material supplementary-material-1] shows the wide strain diversity of *S. halichoeri*.

**Figure 1:**
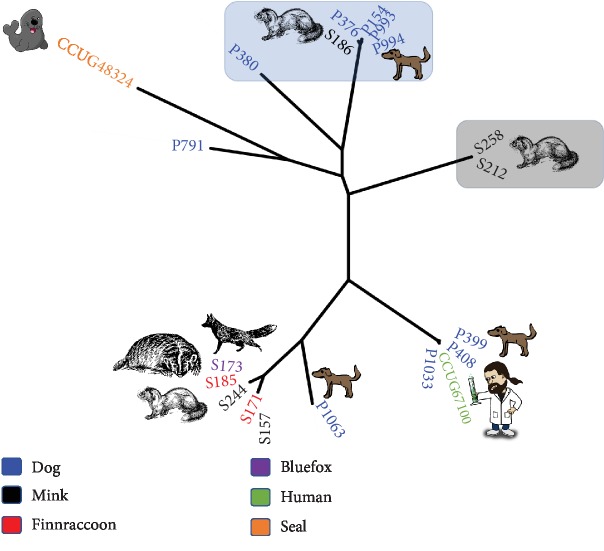
Phylogenetic tree based on core genome (1,456 genes).

A phylogenic tree of 20 *S. halichoeri* strains was constructed using a multiple alignment of 1,456 core proteins as illustrated in Figure 1. Most *S. halichoeri* genomes grouped together into individual clades according to their host-derived origins. Interestingly, there were two separate clades; one was roughly dominated by dogs while the other clade comprised mostly of mink, blue foxes, and Finnraccoons. Similar results were seen by Eklund et al. when partial sequences were compared [9]. The clustering of the dog strains together seems expected, as sharing the same host would likely reflect a same origin for these strains while also niche adaptation could play a role. The grouping of mink-, Finnraccoon-, and blue fox-associated strains was also expected, indicating a common origin of *S. halichoeri* strains in these animals. The human strain (CCUG67100) clustered closely together with three of the dog strains (P399, P408, and P1033) indicating a potential zoonotic connection. It is noteworthy however that the human strain is from the United States of America and the dog strains are all from Finland. Another interesting finding was that the seal strain (CCUG48324) was somewhat different from all the other *S. halichoeri* strains. Most of the mink-associated strains were scattered in different clades except for two strains (S258 and S212), which formed a separate, distinct clade suggesting that there has been more than one introduction of this bacteria into the fur animal community. Interestingly, the dog strain P791 did not cluster with any of the clades and when investigating this further, it was found that this dog came from a different geographic area (eastern Finland) than all the other dogs which were all from the Uusimaa region (south) of Finland. There is also a single mink strain which groups together with the dog strains. Dogs and mink do have some contact within farms so direct transmission is not impossible although likely rare.

### 3.3. Pan, Core, and Accessory Genomes of 20 *S. halichoeri* Strains

**Figure 2:**
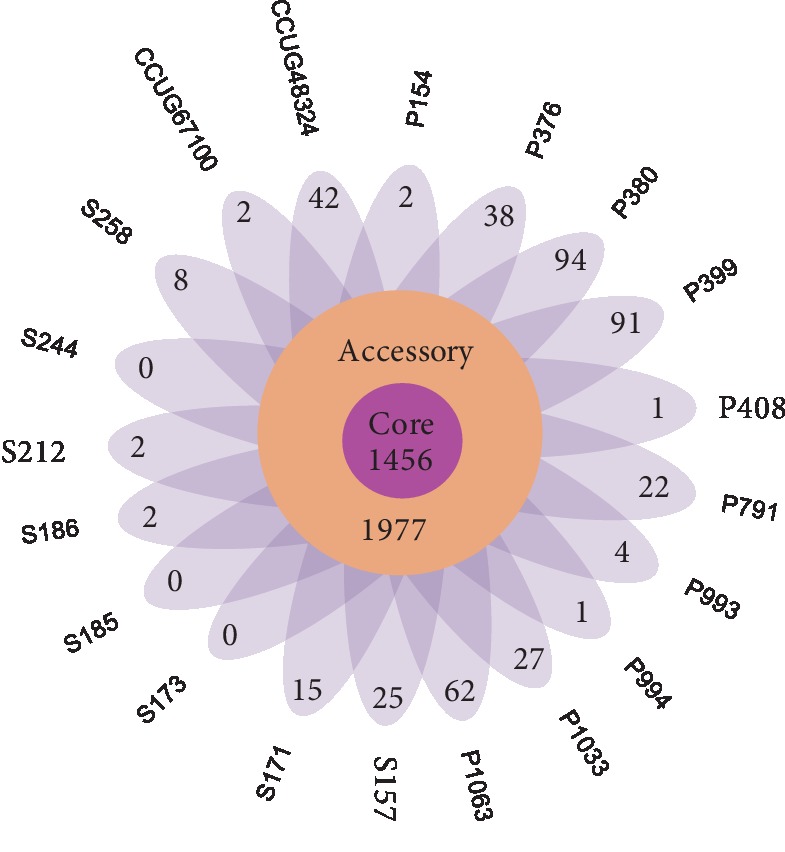
*Streptococcus halichoeri* pangenome (3,433 genes) representing the individual strain-specific genes.

**Figure 3:**
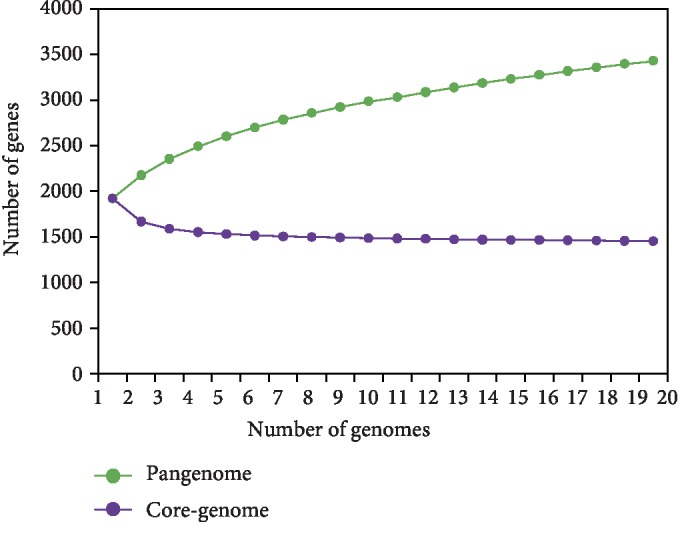
Pangenome development plot of *S. halichoeri.*

We used the genome sequences of 20 *S. halichoeri* isolates to construct the pangenome. The numerous genetic loci from the pangenome essential and necessary for the survival of the bacteria is the core genome of a particular species. These genes are largely involved with different metabolic, catabolic, transport activities and degradation of nucleic acids, ribosomes, and proteins essential for basic housekeeping functions [22, 23]. As a group, these 20 genomes yielded a pangenome of 3,433 genes ([Supplementary-material supplementary-material-1]), of which only 42% (1,456 genes) formed the core genome ([Supplementary-material supplementary-material-1]), revealing a slightly high interspecies diversity (Figure 2) [24–28]. When the number of genes in pangenome was plotted against the number of *S. halichoeri* genomes using Heap's Law calculation (Tettelin et al., [22, 23]), the obtained *α*-value of 0.81 indicated that the pangenome is still open (Figure 3). In a detailed examination of the pangenome development data, it was noticed that the pangenome curve starts to level at approximately 3,000 genes. Genomes added after 13^th^ genome contribute only few genes to the pangenome implying pangenome of *S. halichoeri* is eventually proceeding to a closed status. Addition of a few more strains would eventually close the pangenome representing the entire genetic repertoire of *S. halichoeri*. Similar trends were observed with the core genome development plot (Figure 3) with fewer gene reduction from the core genome after the 8^th^ genome. The comparatively low number of core genes (1,456) in *S. halichoeri* species indicates a broad genome structure, suggesting a large accessory genome. Even with likely possibility of moderately growing pangenome, *S. halichoeri* are undoubtedly a dynamically evolving species with multiple habitats.

The part of *S. halichoeri* pangenome which is not included in the core genome is generally referred to as an accessory genome. These genes are apparently not essential but can provide reasonable advantages to different strains of this species. Accessory genome basically outlines the diversity of the *S. halichoeri* species [22, 23]. The 20 *S*. *halichoeri* strains encompass an accessory genome of 1,977 genes with 438 genes (Figure 2) belonging to strain-specific genes that can only be found in one strain of *S. halichoeri* but absent in all other strains (also called unique genes). The numbers of unique genes per each genome are indicated in Figure 2. Two of the *S. halichoeri* strains (P380, P399) with the highest numbers of contigs (70, 244) could have many partial/split genes which can inflate the count for unique genes in these strains. Interestingly, majority of the dispensable genes of *S. halichoeri* were annotated as hypothetical proteins or proteins with an unknown function ([Supplementary-material supplementary-material-1]), and as most variations exist with unknown and uncharacterized functionalities, it is very problematic to associate any type of adaptive role or benefits for the *S. halichoeri* strains. Nevertheless, in a small number of strains, their unique genes were annotated with a selection of predicted functions from transport and metabolism to phage-related proteins, transposases, and mobile elements.

### 3.4. Virulence

The bacterium is able to jump species and adapt with ease to new environments. This in addition to pathogenic potential could be explained through virulence factors. We were able to identify 19 different streptococcal virulence factors in the core genome. These belong to 5 categories based on their mechanism. These factors are listed in Table 2. The category most represented in the core virulence factors is adhesion-associated products followed by proteases and toxins. These would benefit bacteria inhabiting skin and mucosa and also contribute to necrotizing infection. We identified a non-streptococci-specific factor, multidrug resistance gene which helps bacteria fight host-derived antibacterials and hormones as well as some antibiotics [29]. We further identified a core protein capsule biosynthesis protein capA which is a suspected virulence factor and enables the bacteria to survive in high salt concentrations [30]. This protein is especially interesting as it would support the hypothesis that this bacterium is of marine origin. The core genome also carries two separate antigen A genes which are used in diagnostics and vaccines in other bacteria and may also have immunomodulatory or evasive functions [31, 32]. Interestingly, the strains also had hemolysin and catalase genes despite the original isolates testing catalase negative. When studying the dog and fur animal isolates, Eklund et al. found varying degrees of catalase activity [9] and the genomic findings support this. The expression of the gene may be dependent on environmental factors and warrants further study.

Evidence of many potential mobile genetic elements (MGE) in the genomes was also noted. Several operon-like clusters related to phages were found throughout the genomes. This suggests phages act as transporters of important genes between bacterial hosts. The presence of plasmids, phages, and integrative conjugative element (ICE) indicates the possibility of lateral gene transfer. Other streptococcal species have been found to have similar attributes, most notably *S. canis* which is the closest genetic relative of *Streptococcus halichoeri* [33].

The accessory genome had a further eight virulence factors. These did not correlate between the different host species of the isolates except the agglutinin receptor, also adherence enabler, which was absent only in the human strain but present in all the others. These virulence factors are listed in Table 3. Streptococcal pyrogenic exotoxin A (SpeA) was only present in one strain P380. Equally interesting is the absence of pili-associated genes. Pili are common in pathogenic streptococci and assist with adherence. *S. halichoeri* had multiple adherence genes but not this very common one. Pili also enable motility and are especially found in intestinal bacteria which may suggest another niche for *S. halichoeri.* Earlier results by Eklund et al. showed antibiotic resistance to erythromycin, clindamycin, and tetracycline in selected dog strains and tetracycline resistance in two mink strains. The core genome had only one antibiotic resistance gene patB, but the pangenome had further three ermB, tetO, and inuC. The ermB is often found in streptococci and is known to code for erythromycin and clindamycin resistance. Tetracycline resistance is more commonly coded by the tetM gene, but tetO is also found. All of these are usually in transposons so would possibly be found in the missing parts of the current genomes.

Interestingly, the majority of these well-established streptococcal virulence factors were found in the core genome despite it representing less than half of the genes in a given isolate. This highlights the importance of these genes to the survival of the species. More virulence factors and putative factors can be found in the genome especially as we learn more about the hypothetical proteins within.

## 4. Conclusions

We find that *S. halichoeri* is a highly variable species with several virulence factors which suggest potential for significant pathogenicity. This is supported by the relatively severe human cases as well as the data on the seal and badger isolates. The many varieties of tissue, host selection, and geographic diversity suggest a diverse niche wherein the potential for lateral gene transfer gives way for a rapid adaptation to new growth environments. The core genome is saturated, but the fact that the already large dispensable genome is still somewhat incomplete suggests we have yet to see the full potential of this bacterium's adaptability and host species flexibility.

We found very little host species-specific markers in the genomes but rather loose clustering according to species as though adaptation is still incomplete. This suggests the host switches into dogs, humans, and fur animals which were rather recent and ongoing, possibly coinciding with the beginning of the FENP epidemic. Some further analysis of the virulence factors is called for as there are many more not directly associated with streptococci but which could play a critical role in the pathogenesis of this bacterium. Expression studies should also be made to verify the role and activity of these genes.

Genetic factors such as great numbers of adhesins and salt tolerance proteins as well as the fact that the first isolates were from marine mammals suggest this bacterium may have marine origins. This would also correlate well with the known history of FENP pathogen and that *A. phocae* also associates with seals. In the FENP study, *S. halichoeri* was mainly found in mink which are fed with locally caught fish much more than Finnraccoons and foxes. This together with our finding of *S. halichoeri* from a batch of herring would suggest a possible source of transmission. The Finnish dogs are also fed with raw fish occasionally, but we do not know how often or in what quantities so it is difficult to assess the level of risk and potential exposure. Other possible routes of infection may occur between the animals, both dogs and fur animals, especially in crowded farm environments, between a dog and an owner while other routes may not have been yet found. Fish handling by humans alone has been connected with infections by other marine mammal-associated pathogens. On the other hand, the recent isolation of *S. halichoeri* in a clinical sample from a badger with no contact to marine environment [6] suggests the ecological niche of this bacterium may already be much wider and possibly underdiagnosed. This is supported by at least one human finding wherein no contact to marine environment could be shown. The presence of this plausibly pathogenic bacterium in domestic dogs suggests further an opportunity for more zoonotic transfers making it important to alert diagnostic laboratories in both human and veterinary medicine.

Current data is not enough to confirm or rule out any suggested transmission or entry routes and this requires further studies. The pathogenic potential of this bacterium should also be studied more. Altogether, this study shows the great adaptability of *Streptococcus halichoeri* and we are yet to see the full potential of this emerging pathogen.

## Figures and Tables

**Table 1:** A general overview of thirteen *Streptococcus halichoeri* genomes.

Strain	Host source	Infected organ	Geographic region	Year	Genome accession no	Status	Coverage	Contigs	Size (Mbps)	G+C (%)	ORFs	Proteins	Ref.
P154	Dog	Ear	Finland, Uusimaa	2010	WLZC00000000	Draft	112x	42	2.06	41.3	2025	1978	This study
P376	Dog	Ear	Finland, Uusimaa	2012	WLZD00000000	Draft	90x	47	2.07	41.2	2036	1991	This study
P380	Dog	Ear	Finland, Uusimaa	2012	WLZE00000000	Draft	109x	70	2.00	41.4	1991	1953	This study
P399	Dog	Skin	Finland, Uusimaa	2012	WLZF00000000	Draft	204x	244	2.26	41.5	2198	2150	This study
P408	Dog	Ear	Finland, Uusimaa	2012	WLZG00000000	Draft	120x	33	1.91	41.7	1892	1847	This study
P791	Dog	Ear	Finland, Northern Savonia	2012	WLZH00000000	Draft	130x	50	1.93	41.6	1889	1846	This study
P993	Dog	Skin	Finland, Uusimaa	2015	WLZI00000000	Draft	90x	42	2.05	41.4	2019	1973	This study
P994	Dog	Skin	Finland, Uusimaa	2015	WLZJ00000000	Draft	115x	40	2.04	41.4	2006	1961	This study
P1033	Dog	Ear	Finland, Uusimaa	2015	WLZK00000000	Draft	117x	32	2.02	41.1	1992	1956	This study
P1063	Dog	Skin	Finland, Uusimaa	2015	WLZL00000000	Draft	120x	43	1.95	41.7	1919	1884	This study
S157 B-4^∗^	Mink	Eye	Finland, Ostrobothnia	2010	WLZM00000000	Draft	90x	62	2.03	41.3	1970	1937	This study
S171 B-3^∗^	Finnraccoon	Skin	Finland, Ostrobothnia	2010	WLZN00000000	Draft	79x	76	2.02	41.5	1971	1934	This study
S173 B-1^∗^	Blue fox	Eye	Finland, Ostrobothnia	2010	WLZO00000000	Draft	103x	54	1.93	41.7	1882	1859	This study
S185 B-2^∗^	Finnraccoon	Paw	Finland, Ostrobothnia	2011	WLZP00000000	Draft	89x	59	1.94	41.7	1891	1858	This study
S186 B-6^∗^ c	Mink	Eye	Finland, Ostrobothnia	2011	WLZQ00000000	Draft	75x	44	1.98	41.5	1924	1898	This study
S212 B-7^∗^	Mink	Skin	Finland, Ostrobothnia	2012	WLZR00000000	Draft	61x	34	2.00	41.5	1976	1937	This study
S244 B-5^∗^	Mink	Skin	Finland, Ostrobothnia	2013	WLZS00000000	Draft	130x	56	1.94	41.7	1866	1849	This study
S258 B-8^∗^	Mink	Paw	Finland, Ostrobothnia	2015	WLZT00000000	Draft	75x	41	2.00	41.4	1988	1947	This study
CCUG48324	Seal	Lung	UK, Scotland, Inverness	2003	WLZU00000000	Draft	280x	54	1.89	41.6	1873	1829	This study
CCUG67100	Human	Blood	USA, NC, Raleigh	2015	WLZV00000000	Draft	94x	34	1.91	41.7	1877	1849	This study

^∗^B-1-8 are the strain markers used by Eklund et al. [9].

**Table 2:** Virulence factors found in the core genome of *Streptococcus halichoeri.*

Group	Virulence factor
Adherence	Putative choline binding protein
	Fibronectin-binding protein
	Fibronectin/fibrinogen-binding protein
	Laminin-binding surface protein
	M-like protein
	Sortase A, LPXTG specific
	Collagen-like surface protein
	Streptococcal lipoprotein rotamase A

Enzyme	Enolase
	Streptodornase D

Manganese uptake	Pneumococcal vaccine antigen A homolog

Protease	C3-degrading proteinase
	Immunoglobulin G-endopeptidase (IdeS)/Mac/secreted immunoglobulin-binding protein (Sib38)
	Serine protease, DegP/HtrA, do-like
	Streptococcal cysteine protease (streptopain)/streptococcal pyrogenic exotoxin B (SpeB)
	Streptokinase

Toxin	C3 family ADP-ribosyltransferase
	CAMP factor
	Hemolysin III

Immune evasion	Multidrug resistance protein^∗^

Capsid	Capsule biosynthesis protein capA^∗^

^∗^These proteins are additional to the known streptococcal virulence factors.

**Table 3:** Virulence factors found in the accessory genome.

Group	Virulence factor
Adherence	Agglutinin receptor
	Choline-binding protein A
	Antiphagocytic M protein

Enzyme	Phage hyaluronidase
	Mitogenic factor 2

Protease	C5a peptidase

Superantigen	Streptococcal pyrogenic exotoxin A (SpeA)
	Streptococcal pyrogenic exotoxin K (SpeK)

## Data Availability

The genomes constructed in this study have been submitted into the NCBI genome database and can be accessed with the codes indicated in Table 1.

## Supplementary Materials

Supplementary 1 Figure S1: a circular plot of the different strains of Streptococcus halichoeri depicting the core genes, accessory genome, and general characteristics.

Supplementary 2 Table S1: the predicted annotations of Streptococcus halichoeri pangenome.

Supplementary 3 Table S2: the predicted annotations of Streptococcus halichoeri core genome.
