# Supplementary figures and images for: Streptococcus halichoeri: Comparative Genomics of an Emerging Pathogen
Source: Int J Genomics. 2020 Feb 18;2020:8708305. doi: 10.1155/2020/8708305 (PMC7049441; doi:10.1155/2020/8708305)

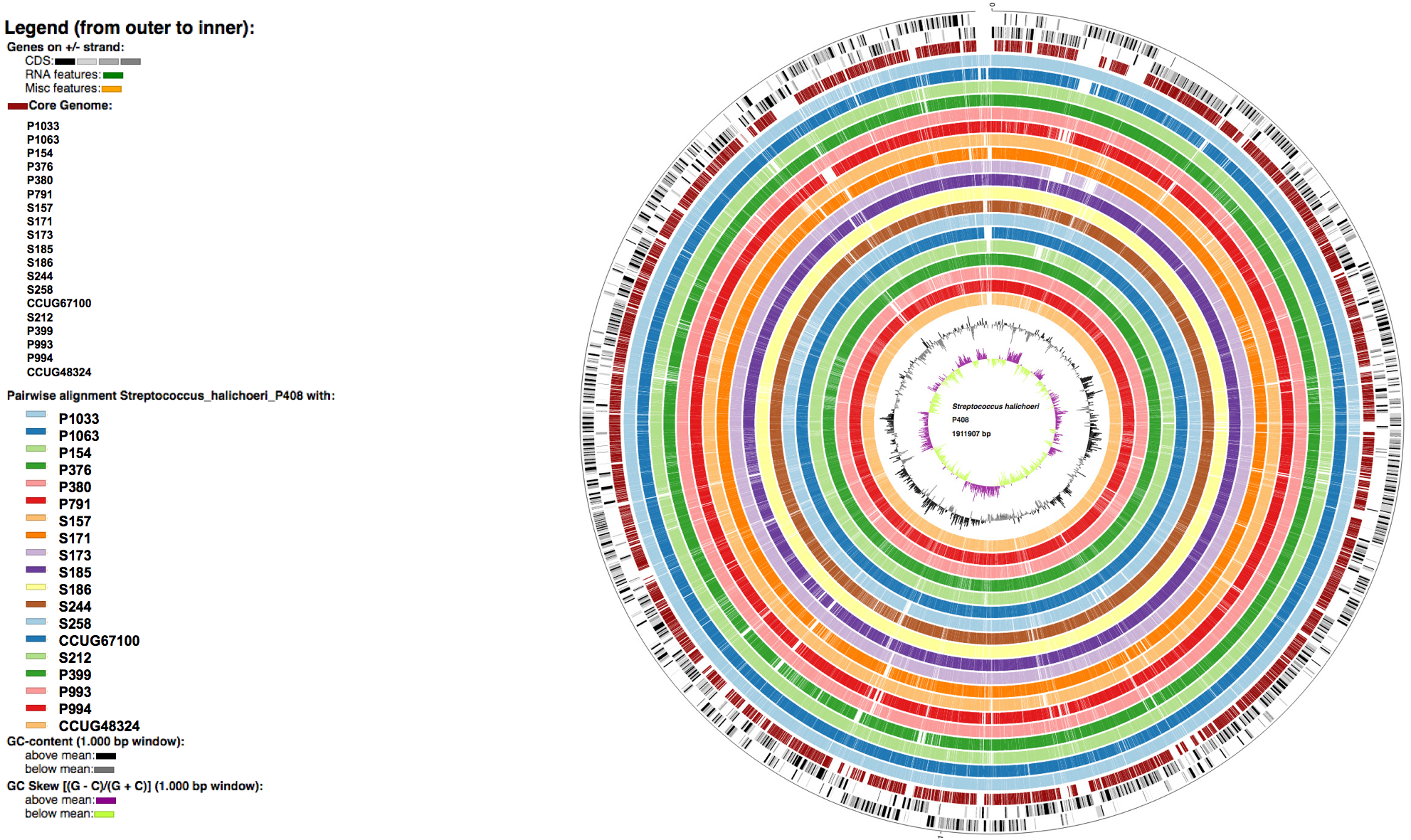

Supplement: Supplementary 1 — Figure S1: a circular plot of the different strains of Streptococcus halichoeri depicting the core genes, accessory genome, and general characteristics. [file 8708305.f1.tif]
